# Pro-arrhythmic effect of escitalopram and citalopram at serum concentrations commonly observed in older patients – a study based on a cohort of 19,742 patients

**DOI:** 10.1016/j.ebiom.2023.104779

**Published:** 2023-08-26

**Authors:** Pari Faraj, Elisabet Størset, Kristine Hole, Godfrey Smith, Espen Molden, Erik Sveberg Dietrichs

**Affiliations:** a Center for Psychopharmacology, Diakonhjemmet Hospital, Oslo, Norway; b Department of Medical Biology, UiT The Arctic University of Norway, Tromsø, Norway; c Institute of Oral Biology, University of Oslo, Oslo, Norway; d School of Cardiovascular and Metabolic Health, University of Glasgow, Glasgow, UK; e Department of Life Sciences and Health, Oslo Metropolitan University, Oslo, Norway; f Department of Pharmaceutical Biosciences, School of Pharmacy, University of Oslo, Oslo, Norway

**Keywords:** Escitalopram, Citalopram, Arrhythmia, Cardiac arrest, Metabolite, Antidepressant

## Abstract

**Background:**

For a decade, patients have been advised against using high citalopram- and escitalopram-doses due to risk for ventricular arrhythmia and cardiac arrest. Still, these drugs are widely used to treat depression and anxiety especially in older patients. It is unclear why they are cardiotoxic and at what serum concentrations patients are at risk for arrhythmias. Thus, how many patients that are at risk for iatrogenic cardiac arrest is unknown.

**Methods:**

We studied the arrhythmogenic effects of citalopram, escitalopram and their metabolites on human cardiomyocytes. Concentrations showing pro-arrhythmic activity were compared with observed drug and metabolite serum concentrations in a cohort of 19,742 patients (age 12–105 years) using escitalopram or citalopram in Norway (2010–2019). As arrhythmia-risk is related to maximum serum concentration, this was simulated for different age-groups from the escitalopram patient material.

**Findings:**

Therapeutic concentrations of both citalopram and escitalopram but not their metabolites showed pro-arrhythmic changes in the human cardiac action potential. Due to age-dependent reduction of drug clearance, the proportion of patients above threshold for arrhythmia-risk increased with age. 20% of patients >65 years were predicted to reach potentially pro-arrhythmic concentrations, following intake of 10 mg escitalopram.

**Interpretation:**

All patients that are using escitalopram or citalopram and have genetic disposition for acquired long-QT syndrome, are >65 years, are using additional pro-arrhythmic drugs or have predisposition for arrhythmias, should be monitored with therapeutic drug monitoring (TDM) to avoid exposure to potentially cardiotoxic concentrations. Serum concentrations should be kept below 100 nM, to reduce arrhythmia-risk.

**Funding:**

This study was funded by The Research Council of Norway (project number: 324062).

**Research in context**

**Evidence before this study**

The U.S. Food and Drug Administration (FDA) have stated that the selective serotonin reuptake inhibitors (SSRI) citalopram and escitalopram increase risk of QTc prolongation and fatal ventricular arrhythmias, like Torsade de Pointes. These recommendations are supported by findings that both drugs increase mortality risk compared to sertraline and paroxetine in older patients. Still, 11% of Norwegian senior residents (>65 years) are prescribed antidepressant drugs, and 35% of these are using escitalopram or citalopram. Both drugs are hepatically metabolized to demethylcitalopram and didemethylcitalopram. Neither of these metabolites are important for the clinical effect but are thought to be electrophysiologically active on the heart. Didemethylcitalopram has been considered most cardiotoxic, due to an association between high serum concentrations and cardiac arrest in dogs but the effect in humans are unknown. There is extensive interindividual variability in the metabolism of citalopram and escitalopram, especially related to age. Such metabolic variability may cause individual differences in cardiac toxicity by citalopram and escitalopram.

**Added value of this study**

We show that therapeutic concentrations of both citalopram and escitalopram have pro-arrhythmic potential on the human cardiac action potential by increasing the triangulation of the cardiac action potential, which is related to increased risk for Torsade de Pointes and cardiac arrest. Contrary to earlier belief, neither of their metabolites appeared cardiotoxic in clinically relevant concentrations. Due to age-dependent reduction of drug clearance, the proportion of patients above threshold for arrhythmia-risk increased with age. 20% of patients >65 years were predicted to reach potentially pro-arrhythmic concentrations, following intake of 10 mg escitalopram. This is increased to 60% after intake of 20 mg escitalopram.

**Implications of all the available evidence**

There is large individual variation in dose-adjusted serum concentrations of escitalopram and citalopram. In general concentrations also increase with age. Many patients therefore reach potentially cardiotoxic concentrations although using recommended doses. Accordingly, patients that are using citalopram or escitalopram and are taking additional pro-arrhythmic drugs, that have underlying genetic disposition for acquired long-QT syndrome, that are >65 years, or have conditions predisposing them for triggering arrhythmias, should be monitored with therapeutic drug monitoring (TDM) to avoid exposure to cardiotoxic concentrations. Serum concentrations of escitalopram (Cmin) should be kept below 100 nM, to avoid pro-arrhythmic effect. Careful cardiological examination should be carried out in risk-patients and use of less cardiotoxic antidepressants than escitalopram and citalopram should be considered.

## Introduction

In 2011, The U.S. Food and Drug Administration (FDA) stated that the selective serotonin reuptake inhibitor (SSRI) citalopram increased the risk of QTc prolongation and fatal ventricular arrhythmias, like Torsade de Pointes (TdP).^1^ Patients were advised against using citalopram-doses above 40 mg and escitalopram-doses above 20 mg.^1^ Risk appeared increased in the elderly, where recommended maximal doses were set to 20 mg and 10 mg, respectively.^2^

In a Canadian study on older patients (mean age 76), both citalopram (the racemate of the (*R*)- and (*S*)-enantiomers) and escitalopram (*S*-citalopram) were found to increase mortality risk compared to sertraline and paroxetine, two other SSRI-antidepressants. Citalopram use also gave a 1.53 relative risk for hospitalization due to ventricular arrhythmia.^3^ Furthermore, patients >65 years obtained 1.4 times higher escitalopram serum concentrations per dose than adults <40 years.^4^ Higher age is also associated with elevated risk of underlying cardiac disease that could be complicated by adverse drug effects. Yet, 11% of Norwegian senior residents (>65 years) are prescribed antidepressant drugs,^5^ and 35% of these are using escitalopram or citalopram.^6^

The high volume of elderly patients being prescribed escitalopram or citalopram advocates a need to estimate the risk for developing ventricular arrhythmias at different drug concentrations. It is therefore important to investigate their electrophysiological effect directly on human cardiomyocytes. Through understanding whether the increased risk of life-threatening arrhythmias is caused by cardiotoxic effects of citalopram and escitalopram metabolites, we can design preventive strategies to reduce risk in predisposed individuals.

Citalopram and escitalopram are both mainly hepatically metabolized to demethylcitalopram and didemethylcitalopram. CYP2C19 is considered the most important metabolising enzyme and genetic variability affects serum concentration of the drugs.^7^ In patients receiving escitalopram, only the *S*-enantiomers of these metabolites are generated while metabolism of citalopram provides both enantiomeric forms. Neither of these metabolites are important for the clinical effect when treating depression and anxiety, and they are usually present in low levels in the circulation. Still, both demethylated and didemethylated metabolites are thought to be electrophysiologically active on the heart,^8^ with didemethylcitalopram considered most cardiotoxic, due to a proposed association between high serum concentrations and cardiac arrest in dogs.^9^ There is extensive interindividual variability in the metabolism of citalopram and escitalopram, partly related to ageing.^5^ Further, some patients appear to have high didemethylcitalopram serum concentrations even after taking doses within the FDA-recommendations.^10^ Such metabolic variability may cause individual differences in cardiac toxicity by citalopram and escitalopram.

**Fig. 1:**
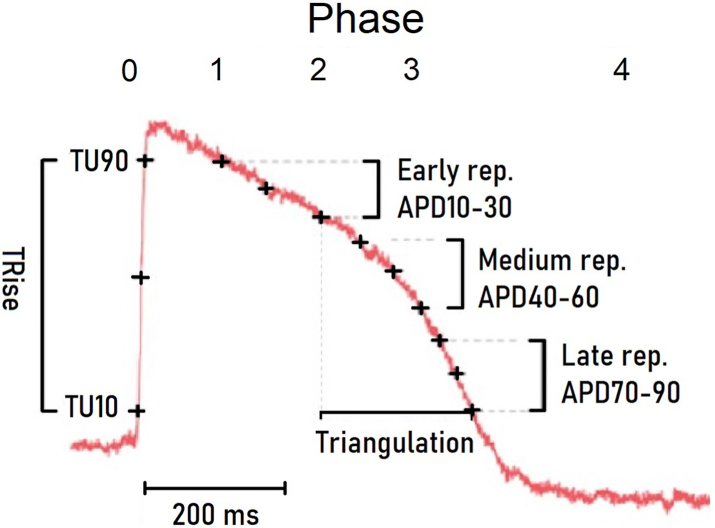
The following (averaged) parameters were obtained from the action potential (AP) recordings of 4 independent replicates: cycle length (CL, Hz); rise time (Trise, ms) between 10 and 90% of the AP amplitude, and AP durations (APD, ms) from 10% to 90% repolarization at 10% intervals. Triangulation was calculated as APD90-APD30.

To characterize the relative pro-arrhythmic potentials of escitalopram, citalopram and their main metabolites, we studied the distinct arrhythmogenic effects of *R*- and *S*-citalopram and their metabolites on the action potential (AP) of human cardiomyocytes, in a well-established model used for cardiotoxic screening of pharmaceuticals.^11^ Due to the known QTc-prolongation risk by citalopram and escitalopram, special emphasis was put on detecting repolarisation disturbances, measured by AP-duration and triangulation of the AP (Fig. 1). Concentrations showing pro-arrhythmic activity were compared with observed drug and metabolite serum concentrations in a cohort comprising 19,742 patients treated with escitalopram or citalopram, categorized by age. As ventricular arrhythmia-risk is most closely related to maximum serum concentration (Cmax),^12^^,^^13^ this was simulated for different age-groups from escitalopram data in the same patient material.

## Methods

### Human cardiac electrophysiology in vitro

#### Effects of *S*-/*R*-citalopram and metabolites on cardiac electrophysiology

##### Human induced pluripotent stem cell-derived cardiomyocyte cell culture

Cryopreserved human induced pluripotent stem cell-derived cardiomyocytes (hiPSC-CM): CDI iCell cardiomycytes^2^ 01434 CardioTox Kit (Catalog #: R1222, FujiFilm CDI, Madison, WI) were kept in liquid nitrogen until culture according to instructions by the manufacturer. The cells have been validated in an international multi-site study^11^ using 28 drugs of varying potency to cause prolonged QT interval and TdP. The sensitivity of individual batches of cells for the present experiment was confirmed by inclusion of 8 wells of a technical standard (3 nM Dofetilide) on each 96 well plate. All studies were conducted at Clyde Biosciences (Glasgow, Scotland). Cells were cultured in 96-well glass-bottomed plates (MatTek, Ashland, Massachusetts) coated with fibronectin (10 mg/ml in PBS supplemented with Ca2+ and Mg2+) (Sigma, St. Louis, Missouri) in a humidified incubator at 37 °C for 3 h. The cell density was 50,000 cells/cm2 (25,000 cells/well). The subsequent maintenance protocols followed manufacturer's instructions using their maintenance medial (FujiFilm CDI Cardiomyocytes Maintenance Media). Experiments were performed between days 4–8. Prior to beginning an experiment, cells were washed in serum-free media (SF media), specifically DMEM (Gibco, ThermoFisher Scientific, UK) supplemented with 10 mM galactose and 10 mM sodium pyruvate.

##### Transmembrane potential signals from hiPSC-CM using voltage sensitive dyes

hiPSC-CM were loaded with 5 μM FluoVolt (ThermoFisher) and the multiwell plate was placed in the CellOPTIQ platform (at Clyde Biosciences Ltd, Glasgow, Scotland). The FluoVolt fluorescence signal was recorded using a 40 × (NA 0.6) objective lens. Excitation wavelength was 480 ± 10 nm, and emitted light was collected by a photomultiplier (PMT) at 510–560 nm. Drugs were supplied by Santa Cruz Biotechnology (Germany) and Toronto Research Chemicals (Canada). Their identity and concentration were blinded from the laboratory personnel. Baseline spontaneous electrical activity was recorded for 20 s prior to drug addition, with only one concentration applied in each well. 30 min after exposure to the tested drug or vehicle a 20 s recording was taken. Offline analysis was performed using proprietary software (CellOPTIQ®). The following (averaged) parameters were obtained from the AP recordings of 4 independent replicates: cycle length (CL, Hz); rise time (Trise, ms) between 10 and 90% of the AP amplitude, and AP durations (APD, ms) from 10% to 90% repolarization at 10% intervals. In this study rate-correction was based on previous studies.^14^ This indicated that over a wide rate of spontaneous rates, there was an approximately linear relationship between the spontaneous cycle length and APD90. This correction was significantly better at correcting for rate-dependent changes than any of the existing QT correction factors.

**Table 1:** Drugs were tested at 3 concentrations (Table 1) with matched vehicle controls for each concentration. A 20 s recording was then taken 30 min after exposure to the drug or vehicle with only one concentration applied in each well.

Substance	Typical concentration (nM)	10× Typical concentration (nM)	100× Typical concentration (nM)
S-citalopram	60	600	6000
R-citalopram	180	1800	18 000
S-demethylcitalopram	15	150	1500
R-demethylcitalopram	20	200	2000
S-didemethylcitalopram	1.5	15	150
R-didemethylcitalopram	6	60	600

##### Tested drug concentrations (Table 1)

Drugs were tested at 3 concentrations with matched vehicle controls for each concentration. The chosen escitalopram (*S*-citalopram) concentration was 60 nM. Assuming no protein binding in the in vitro model, this corresponds to a total serum concentration of 136 nM, as escitalopram protein binding is reported at 55–56% (most commonly 56%, which is used in the present study).^15^, ^16^, ^17^ It is generally accepted that the pharmacological and toxicological effect of a drug greatly depends on its free (unbound) fraction.^18^^,^^19^ This could vary with age and clinical conditions.^20^ According to common pharmacological practice, we have therefore investigated higher concentrations (10× and 100× estimated free fraction in risk patients) of the compounds. This range is clinically relevant according to a recent paper on escitalopram and citalopram intoxications^21^ and allowed us to account for the possibility that the total drug concentration could have cardiotoxic effect. In a large therapeutic drug monitoring (TDM) study 60 nM was the average total concentration in patients with normal escitalopram metabolism through CYP2C19, and 136 nM was below the average total concentration in poor metabolizers.^7^ After administration of racemic citalopram, *R*-citalopram is reported to be 3 times higher than *S*-citalopram in a publication by Ji et al.^22^ The typical concentration of R-citalopram as well as the *S*-/*R*-demethylcitalopram and *S*-/*R*-didemethylcitalopram metabolites, were calculated according to ratios from their paper.^22^

##### Statistical power of APD measurements

Shapiro Wilk tests were performed and confirmed the normal distribution of the APD30 and APD90 parameters prior to statistical testing. Based on knowledge from earlier studies^11^ and pilot data in the same cell model the assay was designed to detect a 10% change in APD90 from untreated baseline, assuming a standard deviation of 6–7% (in our experiment APD90 = 460 ms ± 32 ms). Power calculation with α = 0.05, β = 0.2, power = 0.8 and SD = 6, indicate that with this study design (common DMSO controls and separate drug concentrations), n = 6 is required to detect 10% change in these parameters at any of 4 concentrations.

### Patient serum concentrations

#### Study population

Serum concentration measurements were retrospectively drawn from the therapeutic drug monitoring (TDM) database at Centre for Psychopharmacology, Diakonhjemmet Hospital, Oslo, Norway, to include patients who had performed TDM analysis of citalopram and escitalopram as well as the metabolite demethylcitalopram (presumed to be *S*-demethylcitalopram in escitalopram users) in the period 2010–2019. In a subset of escitalopram users, tested between 2020 and 2021,^23^ we were also able to analyse *S*-didemethylcitalopram. Information regarding age, sex, daily dose and time between last dose and blood sampling was also collected from the database. For patients with multiple TDM measurements, only the last measurement was included. Included patients were divided into four subgroups based on age: (a) < 18, (b) 18–64, (c) 65–80, and (d) > 80 years. Data were not sex/gender-disaggregated as previous studies have not reported clinically relevant differences for the pharmacokinetics of escitalopram.^24^

#### Serum concentration analysis of escitalopram, citalopram and metabolites

The methodological approach to serum concentration measurements of citalopram, escitalopram and metabolites is described in a recent paper.^23^ Measured serum concentrations represent total concentrations, i.e. the sum of bound and unbound drug. In order to relate unbound concentrations that appeared pro-arrhythmic in vitro to the patient data, we derived pro-arrhythmic total serum concentrations, using the previously reported plasma protein binding of 56% for escitalopram.^15^

#### Simulations of maximum escitalopram concentrations

As the standard sampling conditions for escitalopram and citalopram serum concentration for TDM measurements are at trough levels (i.e. 12–24 h after dose), these measurements do not reflect the individual maximum concentrations, which occur about 3–4 h after dose intake representing the true risk of QTc prolongation.^15^ Therefore, modelling and simulation methodology was applied to simulate maximum concentrations of escitalopram in a virtual population.

Simulations were performed using a previously published population pharmacokinetic model of escitalopram.^25^ This model was developed using nonlinear mixed effects modelling to describe escitalopram plasma concentrations as a function of time and dose. The final model was a 1-compartment linear model with first-order absorption, and with age, weight and CYP2C19 genotype identified as significant covariates affecting escitalopram clearance, and body mass index affecting volume of distribution. The interindividual variability in pharmacokinetic parameters was assumed to be log-normally distributed. The residual variability was modelled using a proportional error model with the assumption of a normally distributed random term (Supplemental Figure S1). Simulated datasets consisting of 100 subjects were generated for each of the below-defined adult age groups (i.e. 17–18 years, 18–64 years, 65–70 years and ≥ 80 years), omitting patients <17 years due to uncertainties with extrapolating the population pharmacokinetic model predictions to adolescents and children. The relative covariate distributions for age and CYP2C19 genotype were set equal to the distributions observed in the escitalopram study population. As body weight and BMI were not recorded in the study population, these covariate values were set to the typical values of 76 kg and 27 kg/m^2^, respectively.^25^ For each age group, 100 simulations were performed (i.e. 10,000 subjects in total) at dosing regimens of 10, 20, 30 and 40 mg daily. To summarize the simulation results, the 90% prediction interval (i.e. 5th–95th percentile of simulated maximum concentrations) and the percentage of free maximum serum concentrations above the lowest concentration that significantly increased the triangulation of the AP, was calculated for each age group and dosing regimen.

### Ethics

The study was approved by the Hospital Investigational Review Board and the Regional Committee for Medical Research Ethics (#482562). Ethical approval was obtained without the requirement of patient consent because the study was based on already collected serum samples and existing data retrospectively retrieved from a routine TDM service.

### Statistics

#### In vitro data

Shapiro Wilk tests were used to determine whether the data in this study were normally distributed based on a least-squares fit to a normal function, while Brown–Forsythe test was used to verify homogeneity of variance (Origin version 9, OriginLab Corp., Northampton, Massachusetts). This corresponded with previous studies that also confirmed normality for all included parameters when applied to data from the Fujifilm CDI iCell 2 cell line.^11^ Statistical tests were made using a paired t-tests for differences between absolute values, with P-values adjusted for multiple comparisons using the Benjamini-Hochberg procedure (one-way ANOVA). The relationship between AP duration at 90% of repolarization (APD90) and CL or diastolic interval was fit to single or double exponential functions (Origin version 9).

#### Patient data and simulations

All patients were included unless they met the exclusion criteria. Exclusion criteria were: (a) serum concentration of citalopram, escitalopram and/or demethylcitalopram outside the limits of quantification (LOQ), (b) blood sampling not performed at trough levels, outside the time interval of 10–28 h after last dose intake, (c) prescribed dose of citalopram or escitalopram less/more than 2.5–80 mg or 2.5–60 mg, respectively, and (d) detection of interacting drugs in the citalopram/escitalopram serum sample. In detail, we screened the samples for CYP2C19 inducers carbamazepine, phenobarbital and phenytoin and the CYP2C19 or –2D6 inhibitors bupropion, fluoxetine, fluvoxamine, paroxetine or levomepromazine.

Differences in absolute serum concentrations of citalopram, escitalopram and metabolites as well as differences in time between last blood sampling and daily dose was investigated across the age subgroups using Kruskal Wallis test followed by Dunn's test. P-values were Bonferroni corrected by multiplying the P-value by the number of statistical analyses that were performed. Statistical analyses were performed using IBM SPSS Statistics for Windows, version 28.0 (IBM Corp, Armonk, NY). Figures were made using GraphPad Prism version 9.0.0 for Windows (GraphPad Software, San Diego, CA) or the R software (v. 2.4.1, R Foundation for Statistical Computing, Vienna, Austria). Simulations were performed using the non-linear mixed-effects modelling software NONMEM® version 7.5 (Icon Development Solutions, Ellicott City, MD, USA), with Pirana (v 21.11.1, 100 Overlook Center, Suite 101, Princeton, NJ, 08540 USA) as modelling and simulation interface.

To avoid possible confusion or misinterpretation of our data, we have according to Greenland et al.^26^ not reported results as “significant”. Instead, P-values are included in the figures and tables.

### Role of funders

The funders of this study did not have any role in study design, data collection, data analyses, interpretation, or writing of report.

**Fig. 2:**
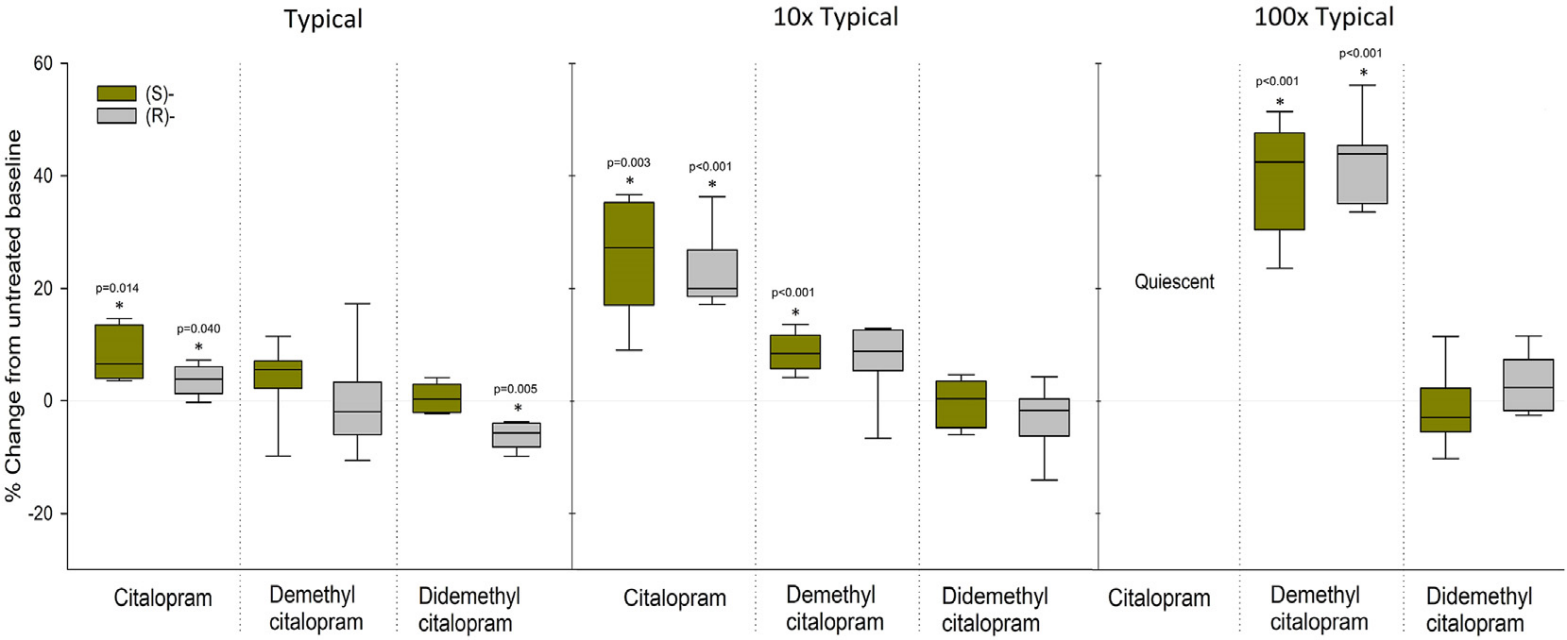
Isolated phase 3 repolarisation was assessed by triangulation (APD90-APD30), which was significantly increased already by low concentrations of S- and R-citalopram, while concentrations of 150 nM S-demethylcitalopram and 2000 nM R-demethylcitalopram were necessary to give a significant increase. No tested concentrations of S-didemethylcitalopram had effect. The box and whiskers show: Maximum: The data point with the highest value below Q3 + 1.5∗IQR (Interquartile range). Upper Quartile: Values contained in the upper 25% of data. Median: The midpoint of the data range. Lower Quartile: Values contained in the lower 25% of data. Minimum: The data point with the lowest value above Q1–1.5∗IQR. Outliers: Values that fall above or below the IQR. Outliers are calculated as is > Q3 + 1.5∗IQR and is < Q1–1.5∗IQR.

## Results

### Electrophysiological effects (Fig. 1, Fig. 2, Supplemental Figures S2 and S3)

Depolarisation (phase 0, see Fig. 1 and Supplemental Figure S2) was measured by TRise. No changes were detected for increasing concentrations of neither R- or S-citalopram, nor metabolites.

Early, medium, and late repolarisation (phase 0–1/2/3, see Fig. 1 and Supplemental Figure S3 for details) was assessed by APD30, APD50 and APD90, respectively. *S*-citalopram: 60 nM increased late repolarisation, while 600 nM reduced early repolarisation. *R*-citalopram: 180 nM increased late repolarisation, while 600 nM reduced early repolarisation. 1800 nM R-citalopram showed a heterogenic effect on the AP: reducing medium and increasing late repolarisation. *S*-didemethylcitalopram: 15 nM increased all repolarisation phases. The effects on medium to late repolarisation were not present at higher concentrations. *R*-didemethylcitalopram: 60 nM increased early repolarisation, while 600 nM increased late repolarisation. *S-demethylcitalopram:* 1500 nM reduced early repolarisation, while late repolarisation was increased. *R-demethylcitalopram:* 2000 nM reduced early but not late repolarisation.

Phase 3 repolarisation (see Fig. 1, Fig. 2) profile is a known independent pro-arrhythmic marker and was studied by assessment of an AP triangulation index (Fig. 1), calculated by APD90-APD30. Triangulation increased at low concentrations of S- and R-citalopram but not their metabolites, in detail: 60 nM S-citalopram and 180 nM R-citalopram increased triangulation, while supratherapeutic concentrations of 150 nM S-demethylcitalopram and 2000 nM R-demethylcitalopram were necessary to give a similar increase. No tested concentrations of S-didemethylcitalopram had a significant effect. 6 nM R-didemethylcitalopram reduced triangulation.

Cycle length (Supplemental Table S1) was not affected by S-citalopram, R-citalopram, S-didemethylcitalopram, nor R-didemethylcitalopram. Both S- and R-demethylcitalopram gave a limited shortening of cycle length for all tested concentrations.

**Fig. 3:**
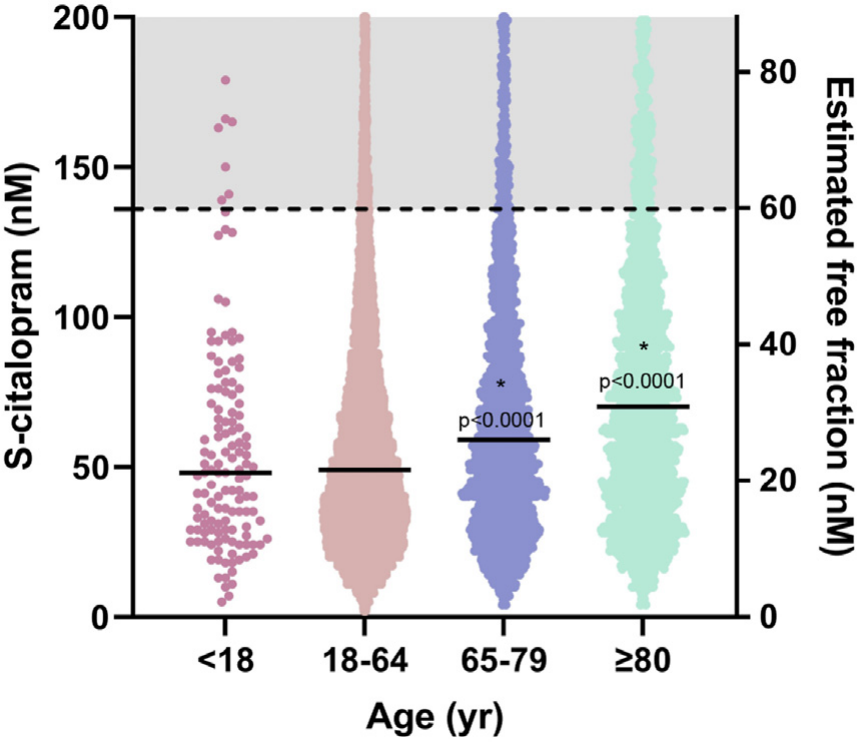
Distribution of S-citalopram in different age groups of escitalopram-users. The stapled line indicates samples that exceed 136 nM, which is the calculated total concentration corresponding to a free concentration of 60 nM, where S-citalopram showed potential pro-arrhythmic activity. 5.4% (n = 130) of the patients <18, 6.3% (n = 13 187) of the patients between 18 and 64, 12.4% (n = 2105) of the patients between 65 and 79 and 15.9% (n = 1933) of the patients ≥80 years old had serum concentrations of S-citalopram exceeding this level. Due to high maximum values (see Table 3), the Y-axis is cut at 200 nM. P-values represent difference from age subgroup 18–64 using Kruskal Wallis test followed by Dunn's test.

**Table 2:** Patient characteristics of included patients divided into four age subgroups.

	Escitalopram				Citalopram			
	<18	18–64	65–79	>80	<18	18–64	65–79	>80
No. of patients (%)	130 (0.8%)	13 187 (76.1%)	2105 (12.1%)	1933 (11.1%)	8 (0.3%)	140 (59.0%)	465 (19.4%)	508 (21.2%)
Gender, F (%)	95 (73.1%)	8445 (64.0%)	1390 (66.0%)	1474 (76.3%)	5 (62.5%)	930 (66.1%)	309 (66.5%)	397 (78.1%)
Gender, M (%)	35 (26.9%)	4742 (36.0%)	715 (34.0%)	458 (23.7%)	3 (37.5%)	476 (33.9%)	156 (33.5%)	111 (21.9%)
Age (yr^a^)	17 (16–17)	40 (30–50)	71 (68–75)	86 (83–90)	17 (14–17)	45 (35–54)	72 (68–76)	87 (84–90)
Daily dose (mg^a^)	10 (10–20)	15 (10–20)	10 (10–20)	10 (5–10)	18 (10–20)	20 (20–40)	20 (10–20)	20 (10–20)
Time between last dose and blood sampling (h^a^)	19 (15–24)	23 (15–25)	24 (22–25)	24 (23–24)	17 (14–22)	23 (15–25)	24 (22–25)	24 (23–24)

a Values are presented as median (interquartile range).

**Table 3:** Serum concentrations of escitalopram or citalopram and their metabolites in mean, maximum and minimum values as well as 10–90 percentiles.

	Escitalopram			Citalopram	
	Escitalopram	Demethylcitalopram	Didemethylcitalopram	Citalopram	Demethylcitalopram
Samples (n)	17 355	17,355	405	2387	2387
Mean^a^	66	30	4.63	163	68
Minimum^a^	7	5	0.34	7	5
Maximum^a^	730	329	35.33	733	395
Percentiles^a^					
10%	21	13	0.82	48	23
25%	33	18	1.72	84	35
50%	52	26	3.47	133	55
75%	84	37	5.90	211	87
90%	126	51	9.65	314	130

a Measurements are presented in nmol/L.

### Patient characteristics (Fig. 3, Table 2, Table 3, Supplemental Table S2, Supplemental Figure S4)

A total of 21,188 patients with TDM measurements of escitalopram were screened for potential inclusion. 676 patients were excluded because escitalopram and/or S-demethylcitalopram were below the lower limit of quantification (LLOQ) or above the upper limit of quantification (ULOQ), 2635 were excluded because the blood sampling was performed <10 or >28 h after last dose intake, 13 were excluded because the dose was <2.5 or >60 mg. Patients with known drug-interactions were excluded, as including these would reduce our ability to estimate the age-dependent effect on serum concentrations and thus the pro-arrhythmic effect. Accordingly, 509 patients were excluded because of interacting drugs (bupropion (n = 386), fluoxetine (n = 4), fluvoxamine (n = 3), paroxetine (=4), phenobarbital (n = 14), phenytoin (n = 5), carbamazepine (n = 59), and levomepromazine (n = 34)), leaving a total of 17,355 patients. In addition, from a subset of 441 escitalopram-treated patients included in a recent study,^23^ we were able to analyse didemethylcitalopram.

For citalopram, a total of 2866 patients were screened for potential inclusion in the study. Of these, 50 patients were excluded because citalopram and/or the metabolite was not detected, 350 were excluded because the blood sampling was performed <10 or >28 h after last dose intake, 79 were excluded because of interacting drugs (bupropion (n = 30), fluoxetine (n = 1), phenobarbital (n = 2), phenytoin (n = 5), carbamazepine (n = 27), and levomepromazine (n = 14)), leaving a total of 2387 patients. All phenotypes of CYP2C19 were included in the patient material for both escitalopram and citalopram. No patients were excluded due to genetic variability in CYP2C19 activity.

Patient characteristics are presented in Table 2 and the distributions of observed escitalopram and citalopram serum concentrations across the age groups in Table 3 and Fig. 3. Depending on age, 5.4%–15.9% of escitalopram serum concentrations were above 136 nM, which corresponds to a free concentration of 60 nM (i.e. the lowest concentration that prolonged triangulation and appeared pro-arrhythmic in vitro), assuming 56% plasma protein binding.^15^

In both escitalopram and citalopram users, the time between last dose and blood sampling was slightly longer in the 65–79 and ≥ 80 age groups (median 24 h, interquartile range 22–25 and 23–24 h) compared to the reference group 18–64 (median 23 h, interquartile range 15–25 h).

**Table 4:** Simulated escitalopram maximum concentrations (Cmax) and percentages above 136 nM, corresponding to 60 nM free concentration in the different age-groups.

Age	10 mg daily		20 mg daily		30 mg daily		40 mg daily	
	Cmax^a^ (nM)	Proportion >136 nM	Cmax^a^ (nM)	Proportion >136 nM	Cmax^a^ (nM)	Proportion >136 nM	Cmax^a^ (nM)	Proportion >136 nM
17–18	49 (21–132)	5%	98 (42–263)	28%	147 (63–395)	55%	195 (83–526)	75%
18–64	61 (25–176)	10%	123 (50–352)	43%	184 (75–528)	70%	245 (101–704)	86%
65–79	76 (30–214)	19%	151 (60–429)	57%	227 (90–643)	81%	303 (120–858)	92%
≥80	80 (31–229)	20%	160 (62–459)	60%	241 (94–688)	83%	321 (125–918)	93%

a Values are presented as median (5th–95th prediction interval).

### Simulations of maximum S-citalopram concentrations (Table 4, Supplemental Figure S5)

Table 4 describes the simulated escitalopram maximum concentration (Cmax) distributions and the percentages of simulated Cmax values above 136 nM, across the various age groups and dosing regimens in escitalopram-users. As expected due to age-dependent clearance, the proportions above threshold increased with age. At the maximum recommended dose of 10 mg daily in subjects ≥65–79 years and ≥80 years, 19% and 20% of subjects were predicted with a Cmax above the threshold, respectively. This proportion increased to 57% and 60%, at 20 mg daily. At the maximum recommended dose of 20 mg in subjects 18–64 years, 43% were predicted with a Cmax above the threshold, increasing to 86% at 40 mg daily.

## Discussion

In the present study, we show that therapeutic concentrations of both S- and R-citalopram have a pro-arrhythmic potential when studying the effect on triangulation of the human cardiac action potential.

Our findings stand in contrast to earlier assumptions, as we show that it is the parent compounds, rather than their demethylcitalopram and didemethylcitalopram metabolites, that increased triangulation, a biomarker of pro-arrhythmic status, at therapeutic concentrations. Increased triangulation of the cardiac action potential (Fig. 1, Fig. 2) is an important determinant of pro-arrhythmia, particularly polymorphic ventricular tachycardias, like TdP.^11^^,^^27^ Both escitalopram and citalopram increase TdP-risk^1^ and patients are advised against using citalopram-doses above 40 mg and escitalopram-doses above 20 mg for this reason.^1^ There are accounts of R-citalopram having adverse effects, suggesting that escitalopram (S-citalopram) is more tolerable than racemic citalopram.^28^ Our study indicated only small differences in electrophysiological effects of R- and S-citalopram, with S-citalopram having a slightly greater effect on AP triangulation as well as prolongating full repolarisation (APD90). A 10 times higher concentration of S-citalopram did however reduce time to early repolarisation (APD30).

Of their metabolites, didemethylcitalopram has been considered most cardiotoxic due to a proposed association between high serum concentrations (39–155 times higher than in humans) and sudden death in dogs.^9^ In our study, we did not find indications that S- or R-didemethylcitalopram are cardiotoxic at therapeutic concentrations. Not even at concentrations comparable to those observed in dogs with sudden cardiac arrest (100 times higher than typical), affected triangulation of the human action potential. This could be ascribed species-dependent differences^29^ or, to speculate, that a more subtle increase in S- and/or R-citalopram, were neglected as potential cause of lethal arrhythmia in the dogs.

Although not posing a considerable risk at therapeutic concentrations, both R- and S-demethylcitalopram gave a dose-dependent increase in triangulation and could be ascribed to an increased risk of TdP in patients with escitalopram- or citalopram-intoxication.^11^^,^^21^ Different from all other tested substances, both demethylcitalopram-metabolites did also affect cycle length, which reduced with between 3.8 and 9.9 percent from baseline for the different concentrations. This could be enhanced in patients with CYP-enzyme variants driving metabolism toward the demethylcitalopram-metabolites.^30^ In the present study, no patients were excluded due to genetic variability in CYP-enzyme metabolism and the material therefore represents a cross-section of the population. We have recently shown that CYP2C19 phenotype largely affects escitalopram serum concentrations.^7^ Reduced metabolism of the drug still seems worse than increased demethylcitalopram-concentration. Both S- and R-citalopram appeared more cardiotoxic, as a significant increase in triangulation occurred already when testing typical serum concentrations (60 nM and 180 nM). Further, a substantial increase was observed at 10 times typical concentrations, while the cells were electrically quiescent after exposure to the highest concentrations (100 times typical), demonstrating the severe cardiotoxicity of these compounds.

The exact ion-channel basis for these effects cannot be determined only from the APD waveform, but there are general considerations which strongly indicate specific ion channels. In the absence of the autonomic stimulation, the primary determinant of the late (phase 3), not early (phase 1/2), sections is the activity of the rapidly inactivating delayed rectifier (I-KI) (aka hERG). Early repolarising phases (1&2) are predominantly affected by L-type Ca channel (I-Ca(L)) and to a lesser extent by the transient outward current (Ito) and the activity of the Na/Ca exchanger (I-NCX). The most common cause of a decrease in the duration of the early phase of the APD as seen in R- and S-citalopram and demethyl-metabolites, is inhibition of I-Ca(L), where moderate inhibition (∼20%) can dramatically decrease APD.^31^ Many compounds are non-selective and bind to multiple ion channels with different affinities, thus complex relationships between APD and concentration can arise, for example verapamil results in shortening of the early phase of repolarisation, while the late phase (phase 3) remains approximately constant as the ADP shortening effect of I-Ca(L) block (phase 1, 2 & 3) balances the prolongation of the phase 3 section of the APD.^32^ This balance of effects results in increased triangulation index (APD90-APD30), promoting TdP.^11^^,^^27^ Similar changes in the AP waveform were observed with R- and S- forms of citalopram and demethylcitalopram but not didemethyl-metabolites of the drugs. This suggests the latter forms of the drug is less active on all ion channel targets in human cardiac muscle.

In the present study, human cardiomyocytes were exposed to S-/R-citalopram and metabolites, that largely were unbound to proteins in the experimental solution. A measured serum concentration of approximately 136 nM would correspond to a free concentration of 60 nM that significantly prolonged AP triangulation. Only 6.3% of the patients in the 18–64 age group had serum concentrations of 136 nM or higher. With old age, albumin levels generally decrease, thereby affecting the free fraction of drugs, but this mainly concerns pharmaceuticals with higher protein binding than escitalopram. Age-related changes in drug elimination are probably of higher concern.^20^ As expected, the fraction of patients with escitalopram (S-citalopram) serum concentrations exceeding 136 nM in our material, increased with age. Among patients between 65 and 79, 12.4% had serum concentrations that increased triangulation in our experiment, while this number reached 15.9% in patients >80 years. Thus, the exposure to potentially cardiotoxic serum concentrations of escitalopram is common in the older patient groups. Further, the serum concentrations included in the present study are sampled 10–28 h after last dose, as therapeutic drug monitoring (TDM) largely is based on measuring the minimum serum concentration (Cmin), due to the higher variability and uncertainty in timing for measuring Cmax. Risk of TdP is however associated with Cmax,^12^^,^^13^ which is reported 3.0 ± 1.5 h (Tmax) after intake for escitalopram,^15^ although our simulations indicated that Tmax may occur later in elderly patients. To assess the potential risk in our patient material, it was therefore necessary to simulate Cmax concentrations from the measured serum concentrations, in the different age groups and for different escitalopram doses.

From our simulation it is apparent that a large proportion of patients, treated with the highest recommended dosage, could be at risk for cardiotoxic effects and TdP. 43% of patients treated with 20 mg escitalopram in the 18–64 age group would have Cmax concentrations exceeding 136 nM. Median concentrations exceeded this level in patients >65 years. In patients at risk, ECG-recordings should be taken at expected Tmax, for best opportunity to reveal pro-arrhythmic changes of escitalopram. According to our findings, there is however chance that only assessing QTc-interval would not reveal risk of TdP. A significant but subtle increase in APD90 (21.9 ms) was observed for the lowest S-citalopram concentration. This corresponds to a QTc-prolongation of only 6.2 ms^33^ and matches the FDA-reported QTc-prolongation (6.6 ms) after intake of 20 mg escitalopram,^1^ showing the strength of our in vitro human cardiomyocyte model. The relative change in triangulation is more profound and could represent a pro-arrhythmic phase 3 repolarisation change in individuals with only a modest QTc-prolongation. The novel ECG-marker QRS/QTc^34^ is a promising marker of TdP-risk in such patients, as it assess late-repolarisation changes relative to depolarisation and early repolarisation changes. It has already proved more accurate than QTc to predict ventricular arrhythmias after escitalopram or citalopram intoxication.^21^

### Caveats and limitations

It is common practice in therapeutic drug monitoring to measure Cmin concentrations, just before next intake of the measured drug. Reference ranges for serum concentrations are therefore based on Cmin concentrations. The rationale for this is that it is far more difficult to predict the time between intake and Cmax values (Tmax), due to individual variations in absorption and elimination. To predict the potential arrhythmogenic effect of the drugs and metabolites at their maximum concentration in the cardiovascular circulation, it was therefore necessary to use a pharmacokinetic model to predict such concentrations from our patient material. It is also impossible to estimate the direct and isolated pro-arrhythmic effect of drugs and their metabolites in patients, as they will be mixed due to continuous metabolism and that measurement of direct cellular electrophysiology is unavailable in the clinic. Electrophysiological assessment of the included substances was therefore assessed in human induced pluripotent stem cell-derived cardiomyocyte, as this was considered our best option.

### Conclusion

We show that escitalopram has a pro-arrhythmic potential when used in therapeutic doses, especially in older individuals where 20% of patients >65 years are predicted to reach potentially pro-arrhythmic concentrations at Cmax, following intake of 10 mg escitalopram. This is increased to 60% after intake of 20 mg escitalopram. Accordingly, all patients that are using additional pro-arrhythmic drugs, or have underlying genetic disposition for acquired long-QT syndrome, that are >65 years, or have conditions predisposing them for triggering arrhythmias, should be monitored with therapeutic drug monitoring (TDM) to avoid exposure to cardiotoxic concentrations of escitalopram. Serum concentrations (Cmin) should be kept below 100 nM, to avoid exposure to a free concentration of S-citalopram associated with pro-arrhythmic changes. Careful cardiological examination should be carried out in at-risk patients and use of less cardiotoxic antidepressants than escitalopram and citalopram should be considered. Patients with known QT-prolongation should avoid these drugs altogether.

## Contributors

Study design: ESD; GS; EM; KH.

Data collection: PF; KH; ES; ESD.

Data analysis and interpretation: PF; KH; ES; EM; GS; ESD.

Writing: PF; KH; ES; EM; GS; ESD.

All authors read and approved the final version of the manuscript. ESD, GS, KH and ES verified the underlying data.

## Data sharing statement

Anonymized data on serum concentrations of the drugs and metabolites, together with a data dictionary will be available upon reasonable request to the corresponding author. Data from the simulations and in vitro human induced pluripotent stem cell-derived cardiomyocyte cell culture model will also be available upon reasonable request to the corresponding author.

## Declaration of interests

The authors report no conflicts of interest.

## Appendix A Supplementary data

Supplemental Tables Percentage **c**hange of cycle length in cells exposed to clinical concentration, 10 x clinical concentration and 100 x clinical concentration of all included substances. n = 112. Statistical analysis was performed using paired t-tests for differences between absolute values, with P-values adjusted for multiple comparisons using the Benjamini-Hochberg procedure. Observed serum concentration of escitalopram across age subgroups in escitalopram users. Percentage of patients with escitalopram serum concentrations exceeding 136 nM, which is the calculated total concentration corresponding to a free concentration of 60 nM, where S-citalopram showed potential pro-arrhythmic activity. P-values represent difference from age subgroup 18–64 using Kruskal Wallis test followed by Dunn’s test.

Supplementary Figure S1 Population pharmacokinetic model used for simulations, derived from (25). The model is a one-compartment model with first-order absorption that predicts escitalopram concentrations (C) as a function of escitalopram dose, time (*t*) and the following pharmacokinetic parameters: absorption rate constant (k_a_), clearance (CL) and volume of distribution (V). Parameters subscripted with TV is the typical value in the population, and η_ka_, η_CL_, η_V_ denote the difference between individual and population values. η_ka_, η_CL_, η_V_ and the residual error ε were assumed distributed with means 0 and variances ω^2^_ka_, ω^2^_CL_, ω^2^_V_ and σ^2^, respectively. The model parameter values were used as previously estimated: K_aTV_ = 0.8 h−^1^, CL_TV_ = 19.8 L/h if CYP2C19 poor/intermediate metabolizer and 26.0 L/h if CYP2C19 normal/rapid metabolizer, Age_median_ = 40 y, θ_age_ = −0.336, V_TV_ = 947 L, ω_ka_=78.9%, ω_CL_=48.5%, ω_V_=62.0% and σ=28.9% (25).

Supplementary Figure S2 Depolarisation (phase 0) was measured by TRise. No significant increase was detected for either R- or S-citalopram, nor their metabolites. The box and whiskers show: Maximum: The data point with the highest value below Q3 + 1.5∗IQR (Interquartile range). Upper Quartile: Values contained in the upper 25% of data. Median: The midpoint of the data range. Lower Quartile: Values contained in the lower 25% of data. Minimum: The data point with the lowest value above Q1–1.5∗IQR. Outliers: Values that fall above or below the IQR. Outliers are calculated as is > Q3 + 1.5∗IQR and is < Q1–1.5∗IQR.

Supplementary Figure S3 Early, medium, and late repolarisation (phase 0–1/2/3) was assessed by APD30, APD50 and APD90. Notably, the intermediate concentrations of R- and S-citalopram caused a shortening of early and medium repolarisation (APD30 and APD50), while late repolarisation was prolonged already by the lowest tested concentrations. The box and whiskers show: Maximum: The data point with the highest value below Q3 + 1.5∗IQR (Interquartile range). Upper Quartile: Values contained in the upper 25% of data. Median: The midpoint of the data range. Lower Quartile: Values contained in the lower 25% of data. Minimum: The data point with the lowest value above Q1–1.5∗IQR. Outliers: Values that fall above or below the IQR. Outliers are calculated as is > Q3 + 1.5∗IQR and is < Q1–1.5∗IQR.

Supplementary Figure S4 Distribution of demethylcitalopram and didemethylcitalopram in the different age groups. Due to high maximum values (see table 3), the Y-axis is cut at 100 nM for demethylcitalopram and 20 nM for didemethylcitalopram.

Supplementary Figure S5 Simulated escitalopram concentration-time profiles at steady state for each age group at the maximum recommended dose specified for the age group of interest. Solid line, median; dashed lines and shaded area: 5th–95th percentile. The dotted line marks 136 nmol/L, corresponding to 60 nmol/L free concentration which significantly increased triangulation of the action potential.

## References

[1] FDA Drug Safety Communication: revised recommendations for Celexa (citalopram hydrobromide) related to a potential risk of abnormal heart rhythms with high doses | FDA. https://www.fda.gov/drugs/drug-safety-and-availability/fda-drug-safety-communication-revised-recommendations-celexa-citalopram-hydrobromide-related.

[2] Selective serotonin reuptake inhibitors: pharmacology, administration, and side effects - UpToDate. https://www.uptodate.com/contents/selective-serotonin-reuptake-inhibitors-pharmacology-administration-and-side-effects.

[3] Qirjazi E., McArthur E., Nash D.M., Reddy H. (2016). Risk of ventricular arrhythmia with citalopram and escitalopram: a population-based study. PLoS One.

[4] Waade R.B., Hermann M., Moe H.L., Molden E. (2014). Impact of age on serum concentrations of venlafaxine and escitalopram in different CYP2D6 and CYP2C19 genotype subgroups. Eur J Clin Pharmacol.

[5] Tveit K., Hermann M., Waade R.B., Nilsen R.M., Wallerstedt S.M., Molden E. (2020). Use of antidepressants in older people during a 10-year period: an observational study on prescribed doses and serum levels. Drugs Aging.

[6] Nasjonalt reseptbasert legemiddelregister – reseptregisteret. http://www.reseptregisteret.no/.

[7] Solhaug V., Haslemo T., Kringen M.K., Molden E., Dietrichs E.S. (2022). Genotyping of patients treated with selective serotonin reuptake inhibitors. Tidsskr Nor Legeforen.

[8] Patel N., Wiśniowska B., Jamei M., Polak S. (2017). Real patient and its virtual twin: application of quantitative systems toxicology modelling in the cardiac safety assessment of citalopram. AAPS J.

[9] Rohrbacher J., Bex P., Gallacher D.J. (2012). Citalopram metabolites inhibit IKs and IKr differentially: is this a possible explanation for the sudden deaths in dogs?. J Pharmacol Toxicol Methods.

[10] Reis M., Chermá M.D., Carlsson B., Bengtsson F. (2007). Therapeutic drug monitoring of escitalopram in an outpatient setting. Ther Drug Monit.

[11] Blinova K., Dang Q., Millard D. (2018). International multisite study of human-induced pluripotent stem cell-derived cardiomyocytes for drug proarrhythmic potential assessment. Cell Rep.

[12] Liu T., Liu J., Lu H.R. (2021). Utility of normalized TdP score system in drug proarrhythmic potential assessment: a blinded in vitro study of CiPA drugs. Clin Pharmacol Ther.

[13] Davies M.R., Martinec M., Walls R. (2020). Use of patient health records to quantify drug-related pro-arrhythmic risk. Cell Reports Med.

[14] Lu H.R., Hortigon-Vinagre M.P., Zamora V. (2017). Application of optical action potentials in human induced pluripotent stem cells-derived cardiomyocytes to predict drug-induced cardiac arrhythmias. J Pharmacol Toxicol Methods.

[15] Rao N. (2007). The clinical pharmacokinetics of escitalopram. Clin Pharmacokinet.

[16] Viatris (2023). https://www.medsafe.govt.nz/profs/datasheet/l/loxalatetab.pdf.

[17] FDA (2009). Highlights of prescribing information - lexapro.

[18] Resztak M., Sobiak J., Czyrski A. (2021). Recent advances in therapeutic drug monitoring of voriconazole, mycophenolic acid, and vancomycin: a literature review of pediatric studies. Pharmaceutics.

[19] Tiula E., Neuvonen P.J. (1986). Effect of total drug concentration on the free fraction in uremic sera. Ther Drug Monit.

[20] Grandison M.K., Boudinot F.D. (2000). Age-related changes in protein binding of drugs: implications for therapy. Clin Pharmacokinet.

[21] Dietrichs E.S., Smith G.L. (2022). Prediction of ventricular arrhythmias by QRS/QTc - ratio in citalopram or escitalopram intoxication. Front Med.

[22] Ji Y., Schaid D.J., Desta Z. (2014). Citalopram and escitalopram plasma drug and metabolite concentrations: genome-wide associations. Br J Clin Pharmacol.

[23] Faraj P., Hermansen A., Molden E., Hole K. (2022). Identification of escitalopram metabolic ratios as potential biomarkers for predicting CYP2C19 poor metabolizers. Ther Drug Monit.

[24] Pastoor D., Gobburu J. (2014). Clinical pharmacology review of escitalopram for the treatment of depression. Expert Opin Drug Metab Toxicol.

[25] Jin Y., Pollock B.G., Frank E. (2010). Effect of age, weight and CYP2C19 genotype on escitalopram exposure. J Clin Pharmacol.

[26] Greenland S., Mansournia M.A., Joffe M. (2022). To curb research misreporting, replace significance and confidence by compatibility: a Preventive Medicine Golden Jubilee article. Prev Med.

[27] Hondeghem L.M., Carlsson L., Duker G. (2001). Instability and triangulation of the action potential predict serious proarrhythmia, but action potential duration prolongation is antiarrhythmic. Circulation.

[28] Sánchez C., Bøgesó K.P., Ebert B., Reines E.H., Braestrup C. (2004). Escitalopram versus citalopram: the surprising role of the R-enantiomer. Psychopharmacology (Berl).

[29] Nánási P.P., Horváth B., Tar F. (2021). Canine myocytes represent a good model for human ventricular cells regarding their electrophysiological properties. Pharmaceuticals (Basel).

[30] Rudberg I., Mohebi B., Hermann M., Refsum H., Molden E. (2008). Impact of the ultrarapid CYP2C19∗17 allele on serum concentration of escitalopram in psychiatric patients. Clin Pharmacol Ther.

[31] Hortigon-Vinagre M.P., Zamora V., Burton F.L., Green J., Gintant G.A., Smith G.L. (2016). The use of ratiometric fluorescence measurements of the voltage sensitive dye di-4-ANEPPS to examine action potential characteristics and drug effects on human induced pluripotent stem cell-derived cardiomyocytes. Toxicol Sci.

[32] Aubert M., Osterwalder R., Wagner B. (2006). Evaluation of the rabbit Purkinje fibre assay as an in vitro tool for assessing the risk of drug-induced torsades de pointes in humans. Drug Saf.

[33] Blinova K., Stohlman J., Vicente J. (2017). Comprehensive translational assessment of human-induced pluripotent stem cell derived cardiomyocytes for evaluating drug-induced arrhythmias. Toxicol Sci.

[34] Dietrichs E.S., Tveita T., Myles R. (2020). A novel ECG-biomarker for cardiac arrest during hypothermia. Scand J Trauma Resusc Emerg Med.

