# Supplementary material for: Pro-arrhythmic effect of escitalopram and citalopram at serum concentrations commonly observed in older patients – a study based on a cohort of 19,742 patients
Source: eBioMedicine. 2023 Aug 26;95:104779. doi: 10.1016/j.ebiom.2023.104779 (PMC10474154; doi:10.1016/j.ebiom.2023.104779)
Supplement: Supplemental Tables — Percentage change of cycle length in cells exposed to clinical concentration, 10 x clinical concentration and 100 x clinical concentration of all included substances. n = 112. Statistical analysis was performed using paired t-tests for differences between absolute values, with P-values adjusted for multiple comparisons using the Benjamini-Hochberg procedure. Observed serum concentration of escitalopram across age subgroups in escitalopram users. Percentage of patients with escitalopram serum concentrations exceeding 136 nM, which is the calculated total concentration corresponding to a free concentration of 60 nM, where S-citalopram showed potential pro-arrhythmic activity. P-values represent difference from age subgroup 18–64 using Kruskal Wallis test followed by Dunn’s test. [file mmc1.docx]

**Supplemental table**

**Supplemental table 1.**

| % Change of cycle length from baseline | **Citalopram** | | **Demethylcitalopram** | | **Didemethylcitalopram** | |
| --- | --- | --- | --- | --- | --- | --- |
|  | *(S)-* | *(R)-* | *(S)-* | *(R)-* | *(S)-* | *(R)-* |
| Clinical concentration | 2.14±4.47 | 0.99±7.43 | -4.33±3.45* | -6.45±4.32* | 1.42±3.67 | -1.40±5.04 |
| 10x | -2.06±5.36 | -1.96±5.39 | -3.81±3.22* | -9.34±2.21* | 9.89±8.49 | -1.09±3.49 |
| 100x | Quiescent | Quiescent | -7.22±2.14* | -8.55±6.93* | 0.09±7.78 | -1.67±2.65 |

**Supplemental table 2.**

| **Age** | **n** | **Escitalopram (nM)^1^** | **P** | **% ≥ 136 nM escitalopram** |
| --- | --- | --- | --- | --- |
| <18 | 130 | 48 (29-75) | 1·000 | 5·4% |
| 18-64 | 13187 | 49 (31-78) | - | 6·3% |
| 65-79 | 2105 | 59 (37-98) | <0·0001 | 12·4% |
| ≥80 | 1933 | 71 (42-111) | <0·0001 | 15·9% |
| ^1^Values are presented as median (interquartile range).  P-values were obtained using Dunn’s test and represent difference from age subgroup 18-64. All P-values are Bonferroni corrected. | | | | |
