# Supplementary material for: Pro-arrhythmic effect of escitalopram and citalopram at serum concentrations commonly observed in older patients – a study based on a cohort of 19,742 patients
Source: eBioMedicine. 2023 Aug 26;95:104779. doi: 10.1016/j.ebiom.2023.104779 (PMC10474154; doi:10.1016/j.ebiom.2023.104779)
Supplement: Supplementary Figure S1 — Population pharmacokinetic model used for simulations, derived from (25). The model is a one-compartment model with first-order absorption that predicts escitalopram concentrations (C) as a function of escitalopram dose, time (t) and the following pharmacokinetic parameters: absorption rate constant (ka), clearance (CL) and volume of distribution (V). Parameters subscripted with TV is the typical value in the population, and ηka, ηCL, ηV denote the difference between individual and population values. ηka, ηCL, ηV and the residual error ε were assumed distributed with means 0 and variances ω2ka, ω2CL, ω2V and σ2, respectively. The model parameter values were used as previously estimated: KaTV = 0.8 h−1, CLTV = 19.8 L/h if CYP2C19 poor/intermediate metabolizer and 26.0 L/h if CYP2C19 normal/rapid metabolizer, Agemedian = 40 y, θage = −0.336, VTV = 947 L, ωka=78.9%, ωCL=48.5%, ωV=62.0% and σ=28.9% (25). [file mmc2.pdf]

$$K_a = K_{a,TV}\ x\ e^{\eta_{ka}}$$

$$CL = CL_{TV}\ x\ (Age/Age_{median})^{\theta_{age}}\ x\ e^{\eta_{CL}}$$

$$V = V_{TV}\ x\ e^{\eta_V}$$

$$C\ (t) = \frac{Dose\ x\ k_a}{V\ (k_a - \frac{CL}{V})}\ (e^{-\frac{CL}{V}t} - e^{-k_a t})\ x\ (1 + \varepsilon)$$
