# Supplementary figures and images for: Pro-arrhythmic effect of escitalopram and citalopram at serum concentrations commonly observed in older patients – a study based on a cohort of 19,742 patients
Source: eBioMedicine. 2023 Aug 26;95:104779. doi: 10.1016/j.ebiom.2023.104779 (PMC10474154; doi:10.1016/j.ebiom.2023.104779)

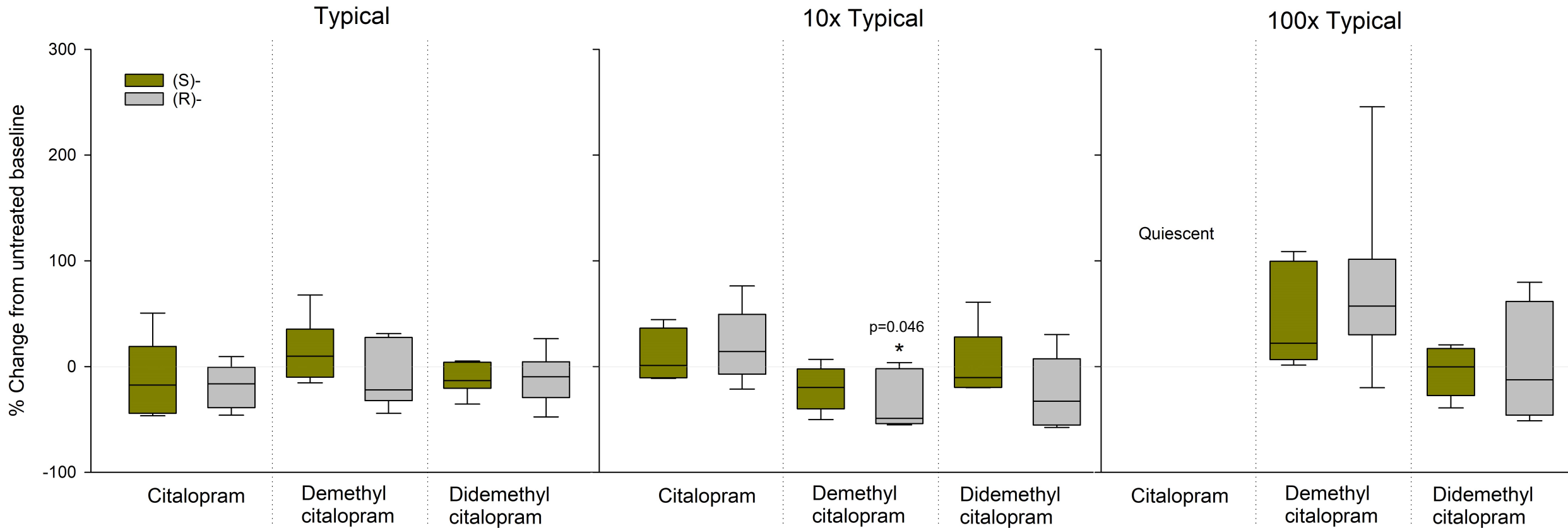

Supplement: Supplementary Figure S2 — Depolarisation (phase 0) was measured by TRise. No significant increase was detected for either R- or S-citalopram, nor their metabolites. The box and whiskers show: Maximum: The data point with the highest value below Q3 + 1.5∗IQR (Interquartile range). Upper Quartile: Values contained in the upper 25% of data. Median: The midpoint of the data range. Lower Quartile: Values contained in the lower 25% of data. Minimum: The data point with the lowest value above Q1–1.5∗IQR. Outliers: Values that fall above or below the IQR. Outliers are calculated as is > Q3 + 1.5∗IQR and is < Q1–1.5∗IQR. [file mmc3.pdf]

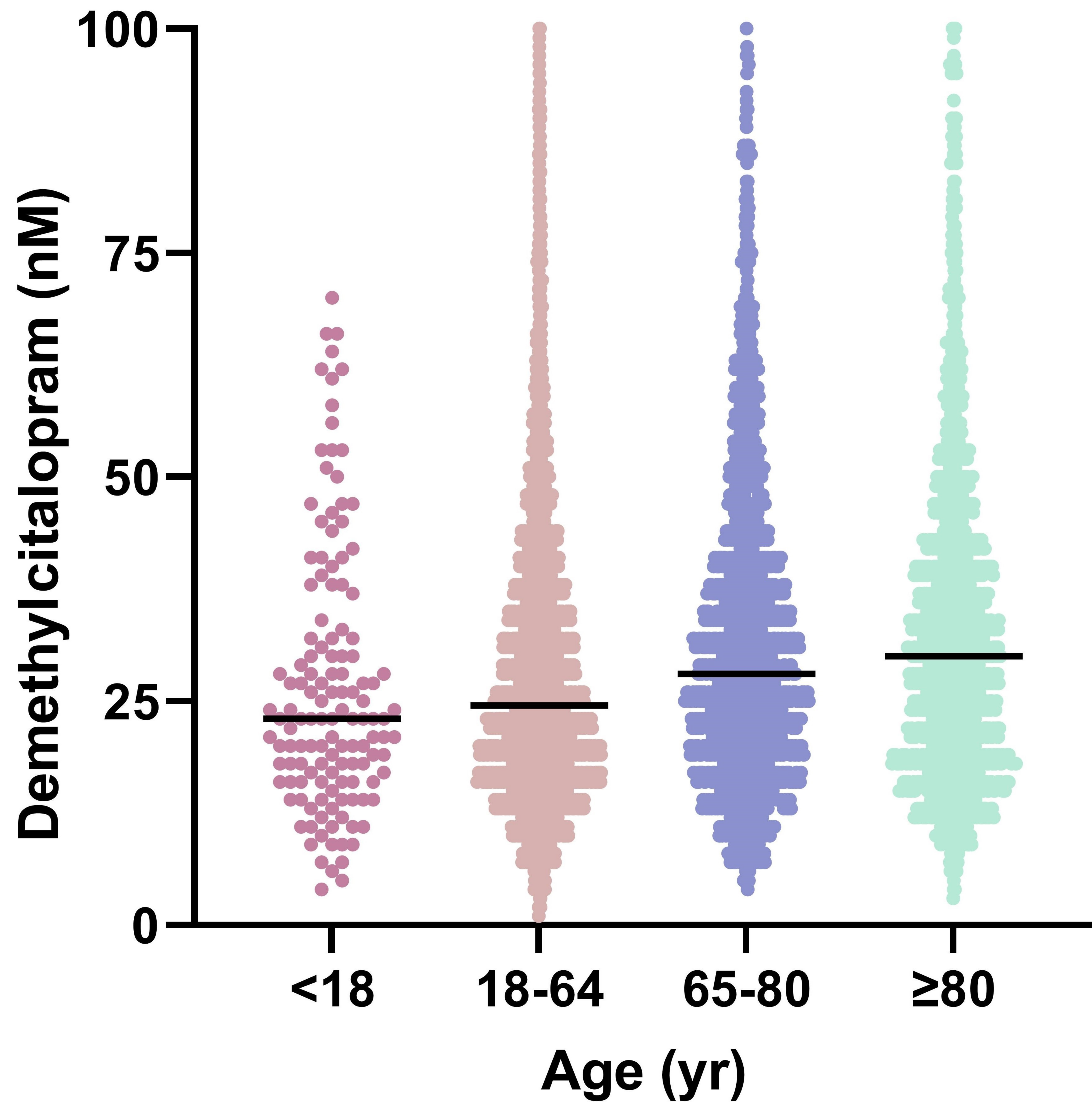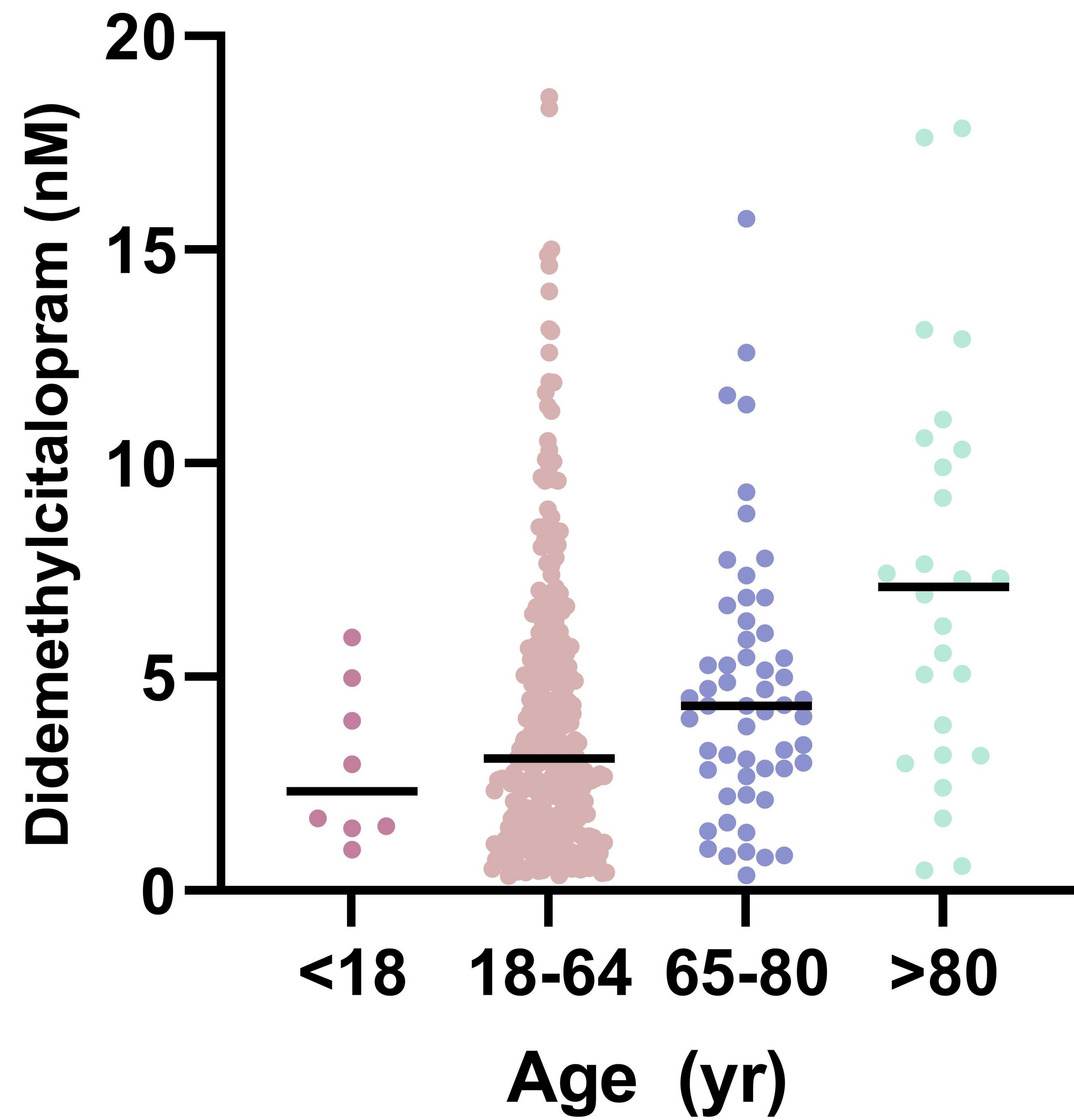

Supplement: Supplementary Figure S4 — Distribution of demethylcitalopram and didemethylcitalopram in the different age groups. Due to high maximum values (see table 3), the Y-axis is cut at 100 nM for demethylcitalopram and 20 nM for didemethylcitalopram. [file mmc5.pdf]

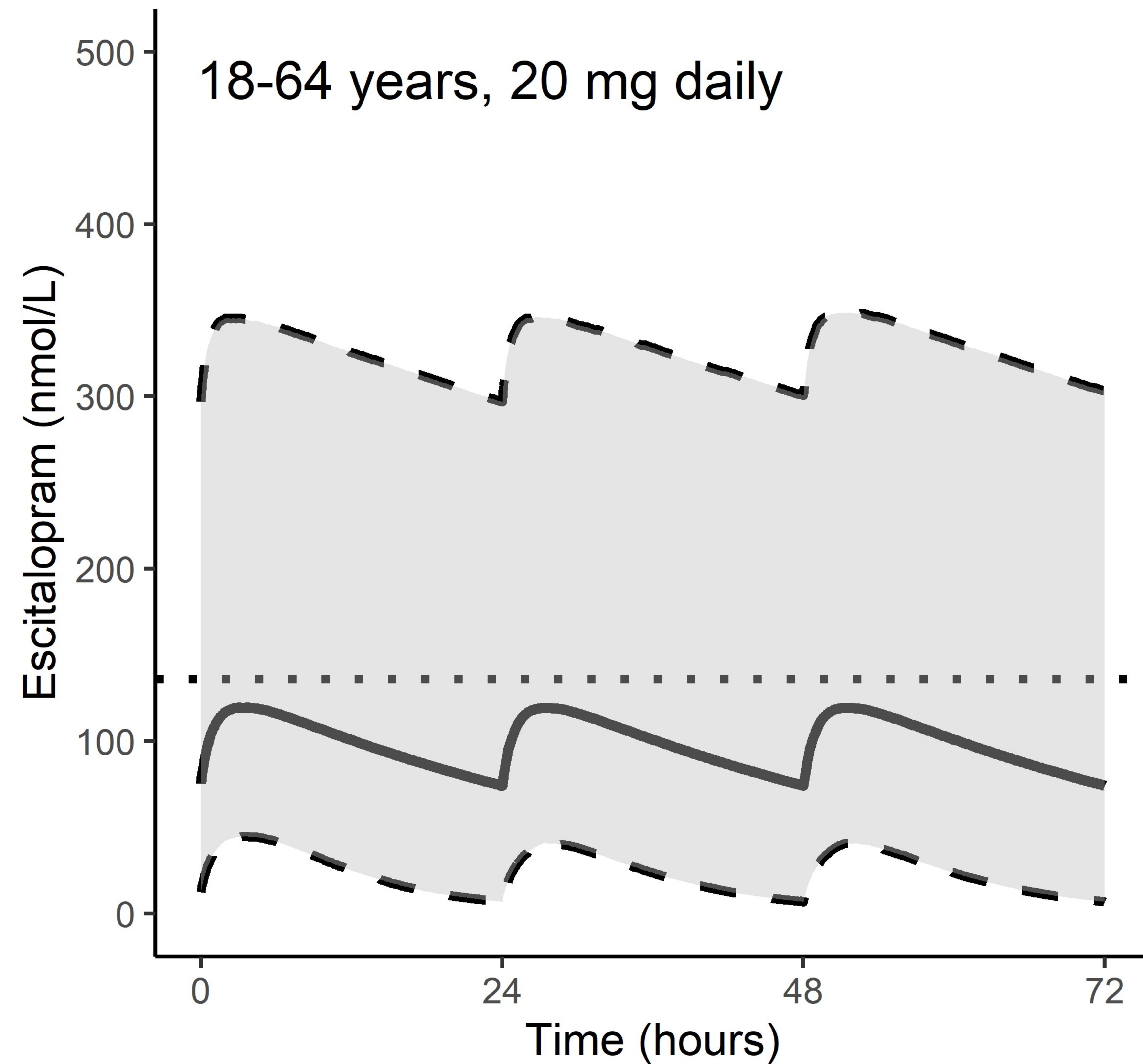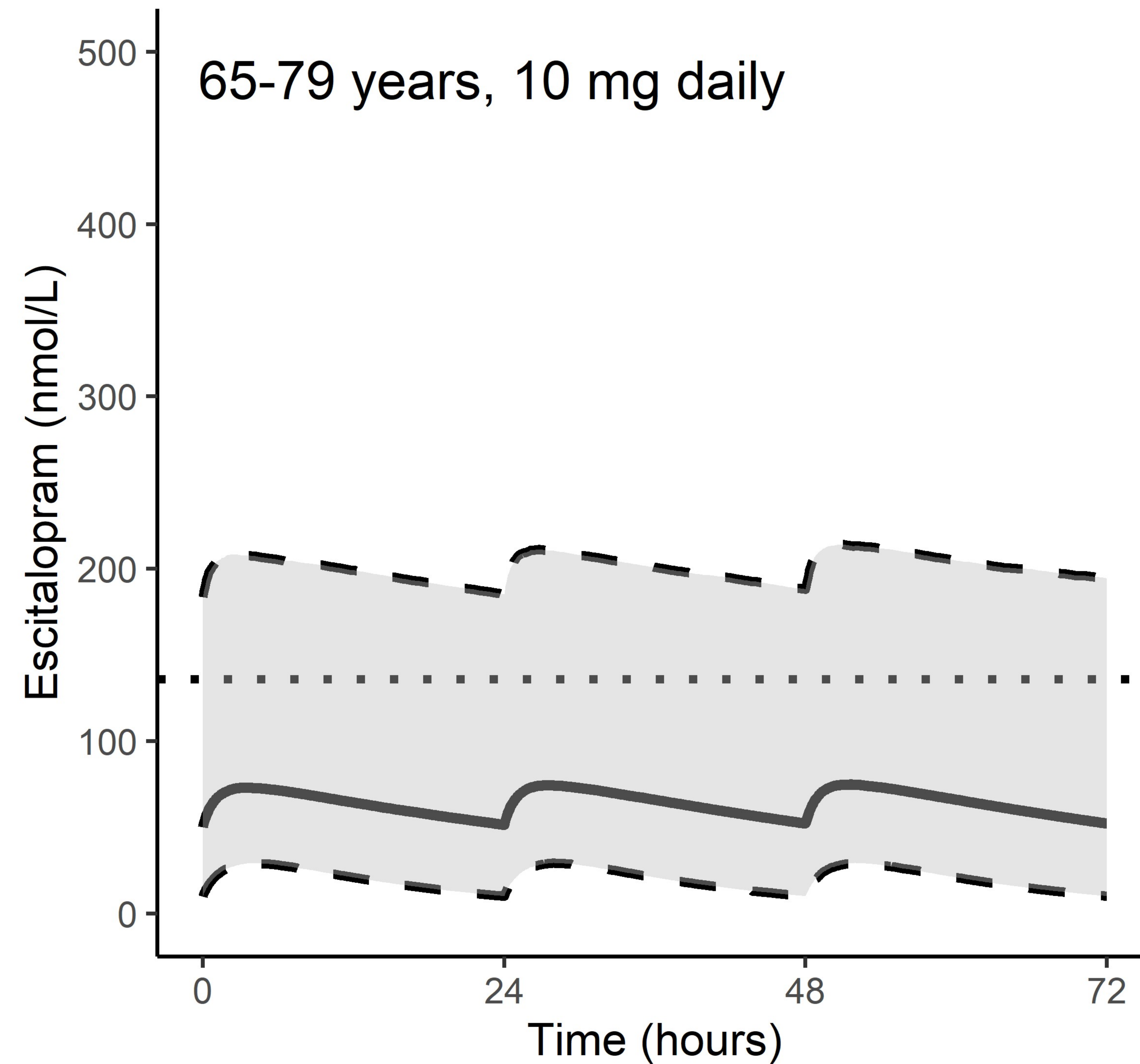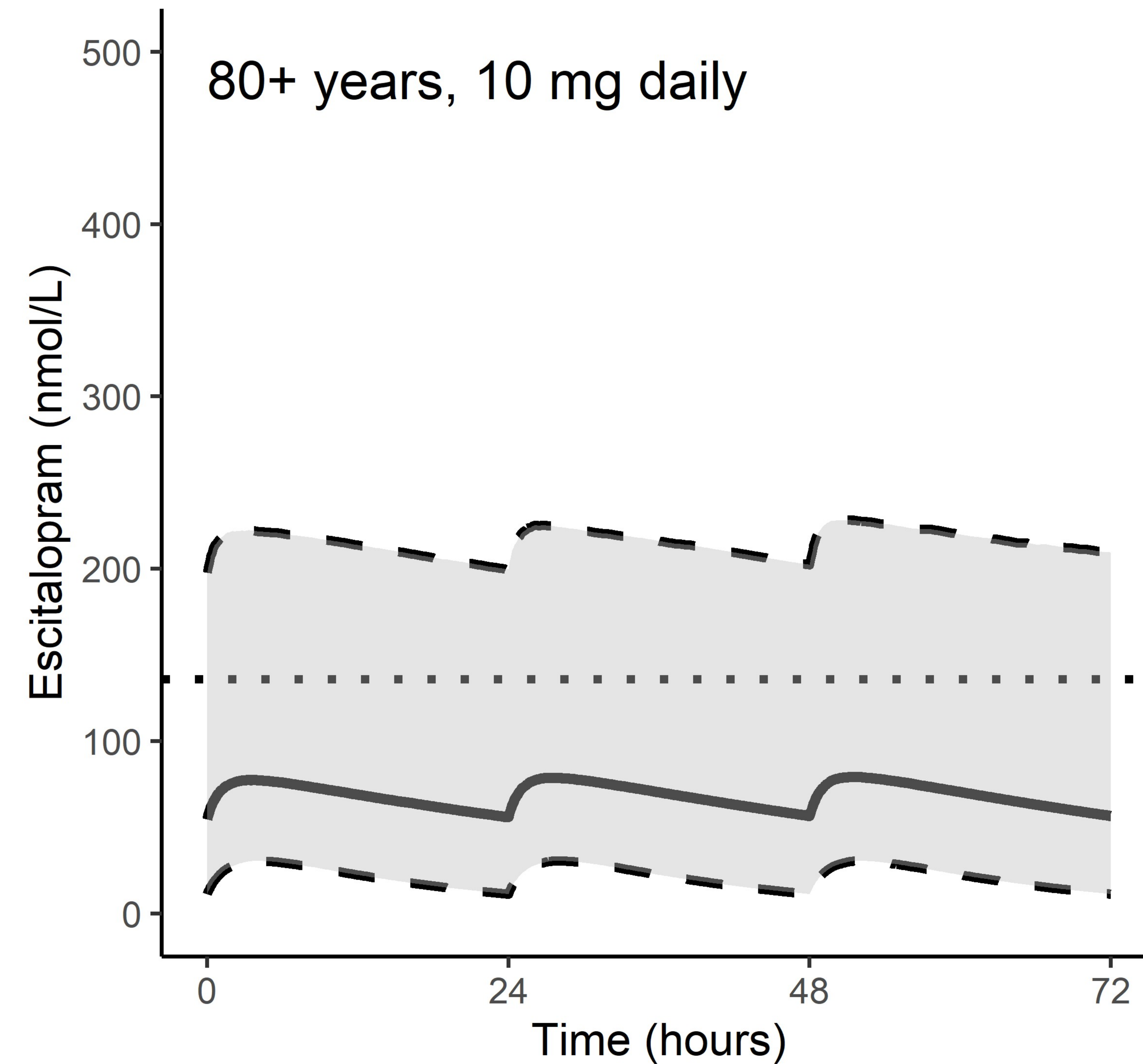

Supplement: Supplementary Figure S5 — Simulated escitalopram concentration-time profiles at steady state for each age group at the maximum recommended dose specified for the age group of interest. Solid line, median; dashed lines and shaded area: 5th–95th percentile. The dotted line marks 136 nmol/L, corresponding to 60 nmol/L free concentration which significantly increased triangulation of the action potential. [file mmc6.pdf]
