# Supplementary material for: Pro-arrhythmic effect of escitalopram and citalopram at serum concentrations commonly observed in older patients – a study based on a cohort of 19,742 patients
Source: eBioMedicine. 2023 Aug 26;95:104779. doi: 10.1016/j.ebiom.2023.104779 (PMC10474154; doi:10.1016/j.ebiom.2023.104779)
Supplement: Supplementary Figure S3 — Early, medium, and late repolarisation (phase 0–1/2/3) was assessed by APD30, APD50 and APD90. Notably, the intermediate concentrations of R- and S-citalopram caused a shortening of early and medium repolarisation (APD30 and APD50), while late repolarisation was prolonged already by the lowest tested concentrations. The box and whiskers show: Maximum: The data point with the highest value below Q3 + 1.5∗IQR (Interquartile range). Upper Quartile: Values contained in the upper 25% of data. Median: The midpoint of the data range. Lower Quartile: Values contained in the lower 25% of data. Minimum: The data point with the lowest value above Q1–1.5∗IQR. Outliers: Values that fall above or below the IQR. Outliers are calculated as is > Q3 + 1.5∗IQR and is < Q1–1.5∗IQR. [file mmc4.pdf]

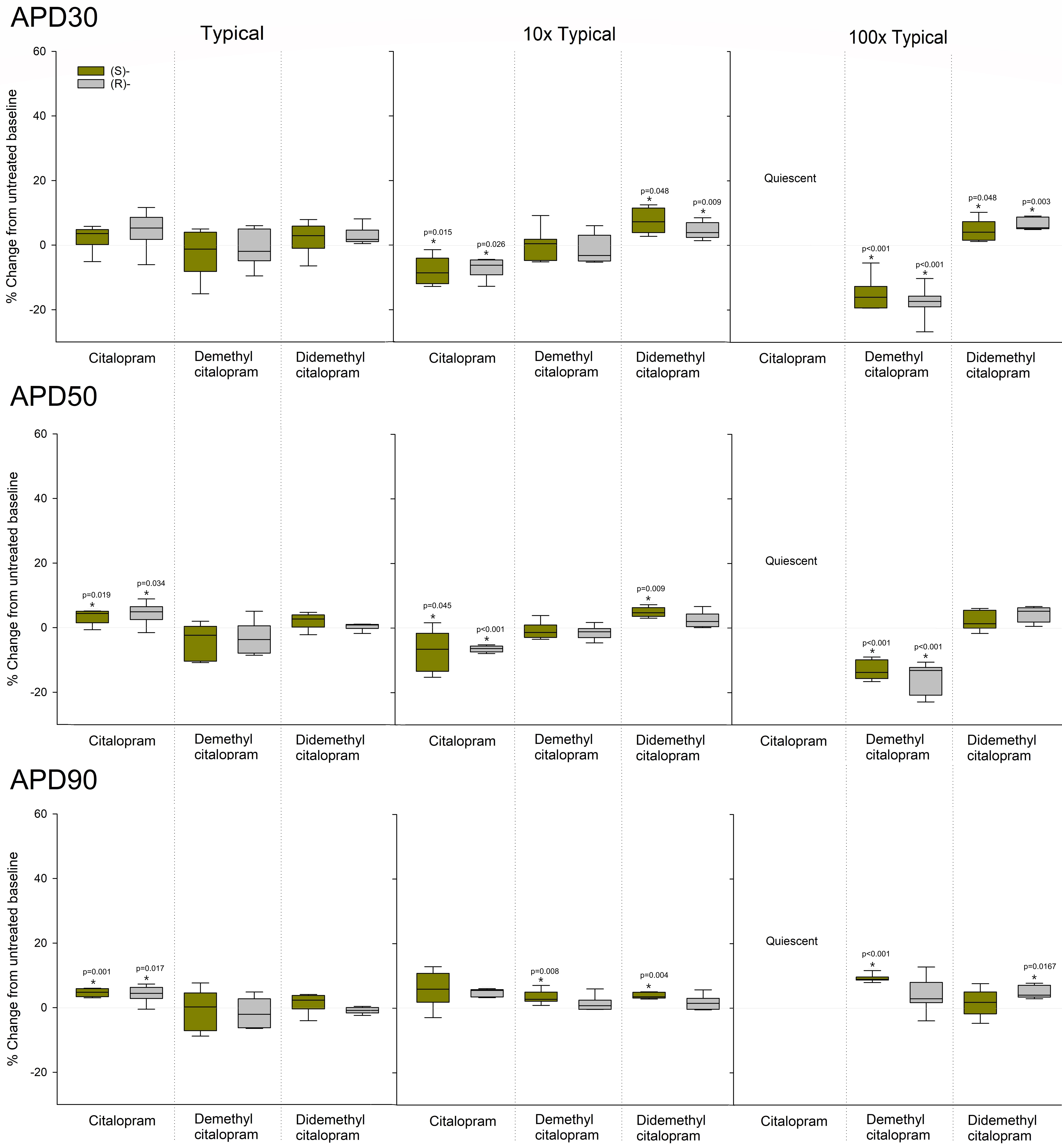

APD30
Typical
10x Typical
100x Typical
(S)-
(R)-
% Change from untreated baseline
60
40
20
0
-20
Quiescent
p=0.015
p=0.026
p=0.048
p=0.009
p<0.001
p<0.001
p=0.048
p=0.003
Citalopram
Demethyl citalopram
Didemethyl citalopram
APD50
p=0.019
p=0.034
p=0.045
p<0.001
p=0.009
p<0.001
p<0.001
APD90
p=0.001
p=0.017
p=0.008
p=0.004
p<0.001
p=0.0167
